# Exploration of the Mediating Role of Self-Compassion and Mindfulness on Orthorexia Nervosa and Perfectionism

**DOI:** 10.1177/00332941241256886

**Published:** 2024-06-04

**Authors:** Eliza Kalika, Misba Hussain, Helen Egan, Michail Mantzios

**Affiliations:** Department of Psychology, 1725 Birmingham City University, Birmingham, UK

**Keywords:** Orthorexia nervosa, perfectionism, mindfulness, self-compassion

## Abstract

Orthorexia Nervosa (ON) is characterized by an excessive preoccupation with healthy eating, accompanied by increasingly restrictive dietary practices over time. In light of the increased attention to ON, it is noteworthy that the existing body of research, specifically with regard to mindfulness and self-compassion remains constrained in scope and depth. A total of 151 participants over the age of 18 completed scales measuring Orthorexia, Self-Compassion, Mindfulness, and Perfectionism. The findings revealed that individuals exhibiting high levels of ON tended to have low levels of self-compassion and mindfulness, along with high levels of perfectionism. Furthermore, the results indicated that self-compassion and mindfulness acted as mediators in the relationship between perfectionism and orthorexia nervosa. These findings deepen our comprehension of orthorexia and underscore the role of self-compassion and mindfulness, or their absence, as mediating factors in this context. The implications of these results and potential future directions are discussed.

## Introduction

Healthy diets have become increasingly popular in recent years as means of achieving optimal health and a desire to improve current health or as a preventative measure however these are common underlying features which may lead to orthorexic tendencies (Dunn & Bratman, 2016). Orthorexia nervosa (ON) was first introduced by Bratman in 1997 (Bratman, 2017), who described ON as an obsessive fixation on healthy eating. While healthy eating is a desirable health behaviour for some individuals the drive for healthy eating may include obsessive thoughts, compulsive behaviours and self-punishment and these are symptoms which collectively have been defined as ON (Bratman, 2017). Individuals with orthorexic tendencies focus purely on food quality and purity (Koven & Abry, 2015) and spend significant time planning and preparing meals that adhere to their food rules. Many individuals who display ON eliminate specific food groups which they consider unhealthy (Dunn & Bratman, 2016). Over time, these restrictions become more extreme resulting in the lack of enjoyment of food, as well as potentially resulting in malnutrition and further medical complications (Brytek-Matera et al., 2017; Cena et al., 2018; Dunn & Bratman, 2016).

The clinical recognition of Orthorexia Nervosa (ON) as an eating disorder remains debated despite the proposal of multiple diagnostic criteria; its absence in the DSM-5 is noteworthy (Donini et al., 2004; Koven & Abry, 2015). ON shares characteristics with established eating disorders (Bartel et al., 2020), and research indicates a link between prior eating disorders and ON development, manifesting as a shift from quantity to quality of food obsession, serving as a socially acceptable weight control strategy for those with disordered eating histories (e.g. Hanganu-Bresch, 2019; McComb & Mills, 2019). Considering the common characteristics between eating disorders and orthorexia, it is important to explore factors that have led to the development and maintenance of eating disorders. One of these factors is perfectionism, perfectionism is a construct with multiple components including high personal standards, excessive concern over mistakes, fear of negative evaluations and self-criticism (Frost et al., 1990). It has been extensively researched in the development and maintenance of eating disorders (e.g., Bardone-Cone et al., 2007; Brown et al., 2012; Franco-Paredes et al., 2004; Rand-Giobannetti et al., 2022), as well as treatment of eating disorders (Bardone-Cone et al., 2009; Egan et al., 2011; Goldstein et al., 2014). A recent systematic review has suggested that perfectionism interventions have been shown to reduce disordered eating and Bulimia Nervosa (Robinson & Wade, 2021). One of the main features of ON is the strict adherence to dietary rules and experiencing self-criticism when deviating from these rules (Bratman, 2017; Mathieu, 2005), this aligns with the components of perfectionism outlined earlier (Frost et al., 1990).

Several researchers have investigated ON and perfectionism (e.g., Barnes & Caltabiano, 2016; Miley et al., 2022; Pratt et al., 2023; Yung & Tabri, 2022). Barnes and Caltabiano (2016) found that elevated levels of perfectionism have been associated with greater orthorexic tendencies. These findings were corroborated by Novara and colleagues (2022) who conducted a cross-sectional study investigating perfectionism and orthorexia and found that individuals classified under a high orthorexic group demonstrated higher levels of perfectionism compared to those in a lower orthorexic group, individuals with orthorexic tendencies scored highest on the subscales of personal standards and organisation. Additionally, they found that those higher in the orthorexic group displayed higher levels of depression (Novara et al., 2022) suggesting that individuals could experience compromised social functioning and depressive mood, which is central to ON (Oberle et al., 2020). This suggests that perfectionism influences orthorexia. However, research has also suggested that the relationship between orthorexia and perfectionism is weaker compared to other eating disorders (Bartel et al., 2020). Given that perfectionism is a significant factor in eating disorders, and considering that orthorexia shares similar characteristics with such eating disorders, it becomes essential to explore perfectionism further within the context of orthorexia.

Mindfulness and self-compassion are closely correlated constructs that have been extensively researched in the context of eating disorders, disordered eating and perfectionism (e.g., Braun et al., 2016; Ferreira et al., 2014; Manova & Khoury, 2023; Tobin & Dunkley, 2021). According to Kabat-Zinn (2003), mindfulness is a psychological concept that entails paying conscious attention to both internal and exterior phenomena such as feelings, thoughts, and bodily sensations without passing judgment. Neff (2003a) has defined self-compassion as understanding that suffering and failure are all part of the human experience, with three key components of self-kindness, shared humanity and mindfulness. As these are closely related there are key differences between the constructs, self-compassion is utilised when facing challenges, personal failures, inadequacies and alleviating suffering** (**Neff & Knox, 2017) whereas mindfulness is about all experiences and not just the challenging ones in the present moment (Siegel et al., 2009). In other words, self-compassion measures mindfulness in response to suffering, while mindfulness is a more holistic measurement tool that explores both positive and negative experiences. Also, the self-compassion scale fails to capture the facets of mindfulness, such as the ability to observe, describe, and act with awareness in the present moment. Research on ON, mindfulness and self-compassion is limited with only three studies (Kalika et al., 2022, 2023; Stahler, 2020). Results from these studies demonstrated that self-compassion and mindfulness are negatively correlated to ON suggesting that those with high orthorexic tendencies display lower levels of mindfulness and self-compassion. These results align with research on perfectionism and mindfulness where those who score highly on perfectionism are in the state of mindlessness which prevents them from being aware of the present moment (Flett et al., 2020), suggesting merit in further exploring the link between orthorexia, perfectionism and mindfulness. Furthermore, James and Rimes (2017) showed that students who experienced difficulties with perfectionism and were placed in mindfulness-based cognitive therapy showed higher levels of mindfulness and self-compassion post-treatment. Previous studies (Kuyken et al., 2010; Manova & Khoury, 2023) found mindfulness and self-compassion to act as mediators between perfectionism and social anxiety and depression, which provide empirical support for investigating similar relationships in the context of orthorexia. Social anxiety, depression, impaired social functioning, and impaired mood are mentioned as key concepts that are predictive of orthorexia (e.g., Awad et al., 2021; Baracat et al., 2024; Barlow et al.. Investigating the mediating role of self-compassion and mindfulness is paramount as it allows for a thorough exploration of mental health factors and quality of life, crucial elements in understanding orthorexia (Kalika et al., 2023). This approach recognizes the intricate connections among these variables and their potential impacts on the development or exacerbation of orthorexia. Given the central emphasis on the challenges and human suffering associated with orthorexia, the potential significance of self-compassion, particularly in comparison to mindfulness, in mediating present and future relationships, holds promise for offering practical solutions to individuals exhibiting high orthorexic tendencies.

The primary objective of this investigation was to examine the connections between orthorexia nervosa (ON), mindfulness, self-compassion, and perfectionism. Notably, no prior research has explored the interrelationships among all of these constructs. Based on existing literature, it is hypothesized that ON will exhibit negative correlations with mindfulness and self-compassion (Kalika et al., 2022, 2023), while showing positive correlations with perfectionism (e.g.,Merhy et al., 2023; Miley et al., 2022; Novara et al., 2021). The secondary goal of this study was to investigate the mediating power of mindfulness in the association between perfectionism and orthorexia nervosa. The final objective was to investigate self-compassion as the potential mediator of the relationship between perfectionism and orthorexia nervosa. The complex connections between perfectionism, orthorexia, mindfulness, and self-compassion warrant thorough examination. This study represents the inaugural exploration of these interconnected concepts which will aid in further understanding of orthorexia nervosa.

## Methods

### Participants

The present study looked at the general population in terms of orthorexia nervosa. A total of 224 participants were initially recruited for the study. However, 73 participants who did not complete the entire study were excluded from the final sample. The sample (*n* = 151) for the present study consisted of 116 females, 31 males, 2 prefer to self-describe as non-binary and 2 participants who preferred not to say, who were all adults (18 years- 67 years; *M = 30.47, SD = 10.84* with a mean Body Mass Index (BMI) of *M = 23.40* kg/m2 (*SD = 4.73)*. Correlation with 4 variables based on a power of .8 for medium effect size and set with the significance of .05 comes to a minimum of 118 participants (Cohen, 1992). A total of 111 participants identified as White, 27 as Asian, 5 as Black, 7 as Mixed and 1 as Other, Furthermore, the type of diet was also collected, the sample consisted of 82 Omnivores, 20 occasional omnivores, 16 semi-vegetarians, 6 pescatarians, 10 lacto-ove-vegetarians, 7 lacto-vegetarians and 10 as vegans. Participants were recruited through volunteering sampling by advertising the study on several social media platforms and forums such as Facebook, Instagram, Twitter and LinkedIn. The advertisement on Facebook has been posted in eating groups requesting individuals to participate in the study. Individuals were also recruited through the university’s Research Participation Scheme. Those who participated in the scheme were rewarded with research credits upon completion of the study. Participants were informed via the information sheet that the inclusion criteria for this study required them to be over the age of 18, have good knowledge of the English language and not be diagnosed with an eating disorder.

### Materials

***Demographic information**:* a set of questions designed to collect general information about participants. Participants were required to report their age, gender, ethnicity, weight, height and type of diet e.g., omnivore, occasional omnivore, semi-vegetarian, pescatarian, lacto-ove-vegetarian, lacto-vegetarian, vegan.

#### Orthorexia Nervosa Inventory (ONI)

The scale was developed by Oberle et al. (2020). It is a measure of ON symptomatology which includes 24 items assessing 3 factors of orthorexic behaviours such as impairments, behaviours and emotions. It utilises a 4-point Likert scale with the following responses: 1 (not at all true) to 4 (very true). The higher total score indicates a greater severity of ON, Oberle et al. (2020) has suggested a score of minimum of 72 to indicate orthorexic tendencies. Sample question are “My healthy eating is a significant source of stress in my relationships” and “I follow a healthy diet with many rules”. The Cronbach alpha for the present study was .97 Additionally, the Cronbach alpha was calculated for the subscales; impairments was .94, behaviours was .92 and emotions was .89. Previous studies have looked at psychometrics of this scale indicating good convergent and criterion validity (Oberle et al., 2020; Zagaria et al., 2023) with Messer et al. (2023) utilising this scale for further validation of orthorexia nervosa symptoms.

#### Five-Facet Mindfulness Questionnaire- Short Form (FFMQ)

This is a shorter version of the original 39-item FFMQ. This scale was developed by Baer et al. (2008) and includes 15 items that measure five facets Observing, Describing, Acting with Awareness, Non-Judging and non-reactivity. This scale utilises a 5-point Likert scale with the following responses: 1 (never true) to 5 (always true). A score is combined for each facet of the scale, with no minimum threshold. Sample questions include “I do jobs or tasks automatically without being aware of what I’m doing” and “I find myself doing things without paying attention”. The Cronbach alpha for the present study was .67. Additionally, the Cronbach alpha was calculated for the subscales; observing was .56, describing was .73, acting with awareness .80, non-judging of inner experience was .86 and non-reactivity was .67. The convergent and discriminant validity of the scale was established in previous research (Bohlmeijer et al., 2011).

#### Self-Compassion Scale (SCS)

This is the original 26-item SCS, it was developed by Neff (2003b) to measure self-compassion. The items are rated on a 5-point Likert scale with the following responses, 1 (never) to 5 (always). This scale includes three compassionate components and three uncompassionate components; the uncompassionate components are reversed scored, these components are self-kindness, self-judgement, common humanity, isolation, mindfulness, and over-identification. Sample questions include “When I fail at something important to me I become consumed by feelings of inadequacy” and “I try to be loving towards myself when I’m feeling emotional pain”. The Cronbach alpha for the present study was .93. Additionally, the Cronbach’s alpha was calculated for the subscales; self-kindness was .83, self-judgement was .84, common humanity was .81, mindfulness was .75 and over-identified was .71. This scale is a valid measure of self-compassion as research indicates strong predictive validity such as group validity (Neff, 2003b, 2015; Neff & Pommier, 2013) and good convergent validity (Neff et al. 2007).

#### Frost Multidimensional Perfectionism Scale (FMPS)

This scale was originally developed by Frost et al. (1990), however, the current study is looking at Stöber (1998) version which contains four subscales instead of six. There are 35 items measuring perfectionism. The subscales are Concern over Mistakes and doubts about actions, Excessive concern with parents' expectations and evaluations, Excessively high personal standards and Concern with precision, order and organisation. Each item is scored on a 5-point Likert scale ranging from 1 (strongly disagree) to 5 (strongly agree). Sample questions include “My parents wanted me to be the best at everything” and “I am very good at focusing my efforts on attaining a goal”. The Cronbach alpha for the present study was .95. Additionally, the subscales were calculated Concern over Mistakes and doubts about actions had .93, Excessive concern with parents' expectations and evaluations had .93, Excessively high personal standards had .83 and Concern with precision, order and organisation had .88. The validity of the measure was tested by Hewitt et al. (1991) indicating that the scale has good validity.

### Procedure

The study received Ethical approval from the ethical committee of an institution based in the midland region of the United Kingdom. Participants were recruited through social media groups and were encouraged to share the study with their connections. They were provided with information about the study, including the criteria for inclusion and exclusion, and the hyperlink to Qualtrics where they could access the questionnaire. Additionally, the university’s Research Participation Scheme was employed offering research credits to individuals who participated. Participants were given a Participant Information Sheet to read, prior to consenting. Participants consented and created a unique code for identifying data in the event of withdrawal. Participants were asked to complete demographic information, ONI, FFMQ, SCS-SF and FMPS. After completion, participants were provided with a debrief form explaining the objectives of the study and the withdrawal process. The study consisted of a single 20-minute online session.

### Data Analysis

Prior to conducting the analysis of the data, assumptions were tested, although options such as bootstrapping and heteroscedastisity-consistent inference can bypass the necessity for normality and homoscedasticity (e.g., Preacher & Hayes, 2008). Firstly, the data was checked for outliers. Cook’s distance was used, and the range was between 0 and .104 which indicated that there were no outliers. According to Hair et al. (2010) the values between 2 to -2 for Skewness and 7 to -7 for Kurtosis are normal. The assumptions for normality were examined using the Skewness and Kurtosis. Skewness scores for ONI, SCS, FFMQ and FMPS were .82 -.08, -.61 and .05. Kurtosis scores for ONI, SCS, FFMQ and FMPS were -.39, -.62, .94 and -.61. So, the data met the assumption for normality. Multicollinearity was tested using the variance inflation factor (VIF) values, the highest value was 2.7 which is below the value of 5 (Tabachnick & Fidell, 2007) meeting the assumption. Additionally, P-P plots and residual scatter plots supported linearity and homoscedasticity assumptions. Data analysis was conducted using SPSS software (version 25.0; IBM Corp., 2017). Pearson’s bivariate correlations were conducted to assess the associations between Orthorexia (ONI), Mindfulness (FFMQ), Self-compassion (SCS) and Frost Multidimensional Perfectionism Scale (FMPS). (see Table 1).

**Table 1.** Bivariate Correlations Between ONI, BMI, FFMQ, SCS and FMPS and Descriptive Statistics.

	1	2	3	4	*M*	SD
(1) ONI					46.66	19.05
(2)BMI	.001				23.38	4.72
(3) FFMQ	−.427**	.178*			45.56	7.35
(4) SCS	−.470**	.179*	.663**		2.74	.83
(5) FMPS	.490**	.019	−.382**	−.648**	92.40	23.79

*Note.* ONI: Orthorexia Nervosa Inventory. BMI: Body Mass Index. FFMQ: Five-Facet Mindfulness Questionnaire SCS-SF: Self-Compassion Scale. FMPS: Frost Multidimensional Perfectionism Scale.

*Correlation is significant at the .05 level.

**Correlation is significant at the .01 level.

**Figure 1.**
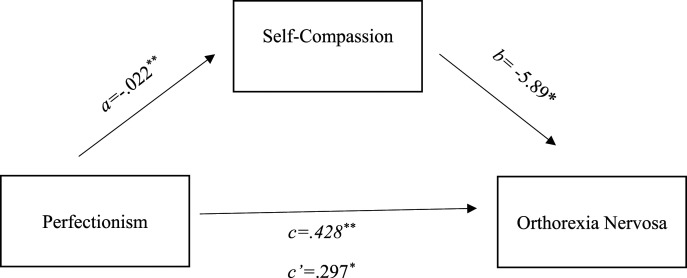
Parallel mediation using standardized regression coefficients to examine the interaction of self-compassion in the relationship between a) Perfectionism and b) orthorexia nervosa. Notes: *a* is the effect of perfectionism on self-compassion; *b* is the effect of self-compassion on orthorexia nervosa; *c* is the effect of perfectionism on orthorexia nervosa; *c’* is effect of perfectionism on orthorexia nervosa with self-compassion in the model.

**Figure 2.**
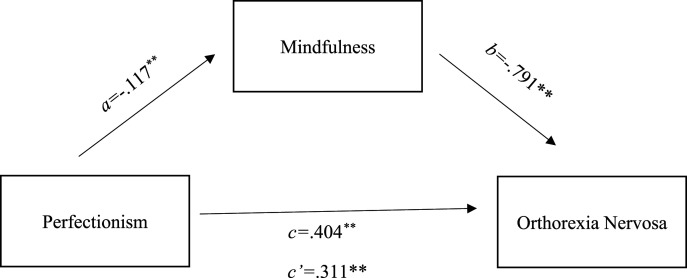
Parallel mediation using standardized regression coefficients to examine the interaction of mindfulness in the relationship between a) Perfectionism and b) orthorexia nervosa. Notes: *a* is the effect of perfectionism on mindfulness; *b* is the effect of mindfulness on orthorexia nervosa; *c* is the effect of perfectionism on orthorexia nervosa; *c’* is effect of perfectionism on orthorexia nervosa with mindfulness in the model.

Furthermore, mediation analysis was used to evaluate the indirect effects (via self-compassion and mindfulness) of perfectionism on orthorexia nervosa (see Figure 1 and 2). Hayes’ (Preacher & Hayes, 2008) PROCESS macro (v3.3) was installed on SPSS (version 25.0) and was used to conduct mediation analyses (model 4) using 10,000 bootstrapping resamples to generate 95% bias-corrected confidence intervals for the indirect effect (Preacher & Hayes, 2008). According to specified guidelines using mediation analyses, Fritz and MacKinnon (2007) suggested that a sample size of 148 participants would enable research to observe an indirect effect of a small-medium sized alpha pathway coefficient (i.e. predictor to mediator) and a small-medium sized beta pathway coefficient (i.e. mediator to criterion) at 80% power using bias-corrected bootstrapping estimating procedures.

## Results

A multiple correlation analysis has been used to identify which scales (BMI, SCS, FFMQ and FMPS) relate to ONI. 

Inter-correlations between ONI, BMI, SCS, FFMQ and FMPS, are presented in Table 1 with r < 0.3 indicating a weak correlation, 0.3 ≤ r < 0.5 indicating a moderate correlation and r ≥ 0.5 indicating a strong correlation (Ratner, 2009). Findings indicate that there are significant negative relationships between ONI and FFMQ (*p* < .001), SCS (*p* < .001) and FMPS (*p* < .001) Only BMI was not significant concerning ONI. In addition, correlational analysis on the subscales of ONI was performed results are presented in Supplementary Table 2 found in supplementary materials. A further correlation analysis has been conducted between the ONI, and subscales of FFMQ, SCS and FMPS, the findings are presented in Supplementary Table 3 found in supplementary materials.

The mediational model analyses (see Figure 1) used orthorexia as the dependent variable, perfectionism as independent variable, and self-compassion as potential mediator. The c indicated a significant relationship between perfectionism and orthorexia *b = .428, p* < .001, 95%*CI* [.309, .548]. Pathway a showed that perfectionism predicted self-compassion *b =* −.022, *p* < .001, 95%*CI* [-.027, −.018], however for pathway b self-compassion did predict orthorexia *b = -*5.89, *p* < .05, 95%*CI* [-10.37, −1.42]. When self-compassion was included in the mediation model, it remained significant *b =* .297, *p* < .005, 95%*CI* [.143, .451], this therefore suggests that the relationship between restrained eating and orthorexia is mediated by self-compassion.

The second mediational model analyses (see Figure 2) used orthorexia as the dependent variable, perfectionism as an independent variable, and mindfulness as potential mediator. The c indicated a significant relationship between perfectionism and orthorexia *b =* .404, *p* < .001, 95%*CI* [.284, .523]. Pathway a showed that perfectionism predicted mindfulness *b =* −.117, *p* < .001, 95%*CI* [-.166, −.069], however for pathway b mindfulness did predict orthorexia *b = -*.791, *p* < .005, 95%*CI* [-1.19, −.389]. When mindfulness was included in the mediation model, it remained significant *b =* .311, *p* < .001, 95%*CI* [.187, .434], this therefore suggests that the relationship between perfectionism and orthorexia is mediated by mindfulness.

## Discussion

This study represents the inaugural exploration of the connections between orthorexia nervosa, perfectionism, mindfulness and self-compassion, with the potential to develop further relevant research and interventions. The results of this study corroborate prior findings demonstrating a consistent negative relationship between mindfulness and self-compassion and orthorexia nervosa (Kalika et al., 2022, 2023; Stahler, 2020). Findings suggest that individuals who display higher levels of orthorexic tendencies display lower levels of self-compassion and mindfulness. These results also align with existing research on eating behaviours and mindfulness, as mindfulness has been linked to lower levels of disordered eating (Beshara et al., 2013; Dutt et al., 2019; Mantzios et al., 2018, 2019; Mantzios & Wilson, 2013, 2014, 2015). Three of the subscales were negatively correlated with ON, these were *non-judgement*, *acting with awareness* and *describing*, which follows recent findings (Kalika et al., 2022). Unsurprisingly individuals with orthorexic tendencies display high levels of judgement as research has shown that those who violate their food rules display elevated levels of distress, self-judgement and self-punishment (Bratman, 2017; Koven & Abry, 2015).

A key concept of self-compassion is self-kindness (Neff, 2003a), the present study has found that the self-kindness subscale had the lowest levels in relation to orthorexia. Having lower levels of self-compassion might indicate that individuals with orthorexic tendencies will not view orthorexic eating as a way of being kind to themselves as research suggests that they engage in their eating as a means of improving their health (which could be viewed as an act of self-kindness. A qualitative study done by Lewthwaite and LaMarre (2022) supports these findings as they have found that orthorexic individuals acknowledged that restrictive eating was not viewed as an act of self-kindness. However, others recognised that having dietary flexibility where they consumed treats and occasional unhealthy foods was an act of self-kindness as it allowed them to become more healthful individuals. This could potentially mean that there is a distinctive difference between healthy orthorexia and orthorexia nervosa proposed by Roncero et al. (2021). Healthy orthorexia refers to non-pathological healthy eating and interest in nutrition, whereas orthorexia nervosa refers to disordered eating that is characterised by obsessive preoccupation with healthy eating (Zickgraf & Barrada, 2021). Therefore, those who display healthy orthorexia could have higher levels of self-compassion and mindfulness as they acknowledge that there needs to be flexibility in terms of eating (Lewthwaite & LaMarre, 2022) whereas those with orthorexia nervosa could potentially have low levels of self-compassion and mindfulness.

Investigating perfectionism was also one of the main aims of this study. The present study reveals that individuals with higher orthorexic tendencies display higher levels of perfectionism. The findings are replicated in accordance with findings in past literature (e.g., Merhy et al., 2023; Miley et al., 2022; Novara et al., 2021). Furthermore, the subscales are also positively correlated with ON except for the *Organisation* subscale, which is no surprise as the *Organisation* subscale is removed from the total score (Stöber, 1998) due to not being problematic and a subscale designed for the assessment of constructive qualities. *Concern over mistakes and doubts subscale* had the highest positive correlation out of all of the subscales followed by the *Excessive concern with parent’s expectations* which is an unexpected finding. High levels of *Concern over mistakes and doubts* have been associated with higher levels of anorexia nervosa and bulimia nervosa (e.g., Boisseau et al., 2013; Bulik et al., 2003) and eating pathology (Davies et al., 2009; Egan et al., 2011). Due to ON having similar characteristics to other eating disorders, this relationship aligns with past literature. Furthermore, individuals with high orthorexic tendencies set themselves strict dietary rules that require them to spend a significant amount of time researching to choose the most appropriate foods according to their rules (Bratman, 2017). If they do not adhere to their rules then the individual experiences self-hatred, self-criticism and guilt (Bratman, 2017; Mathieu, 2005). High scores on *Excessive concern with parental expectations* subscale is an unexpected finding. Research has suggested that ON is mostly influenced by desire to achieve optimal health, improving general health (Bratman, 2017; Dunn & Bratman, 2016) and social media exposure (Turner & Lefevre, 2017). However, a qualitative study done by Cheshire and colleagues (2020) highlighted that parental influences emerged as significant in the development of orthorexia, this could either be the extreme religious beliefs in the family, parental dietary choices or challenging relationships with parents thus expressing themselves through eating. However, the sample of this study included health care professionals and those who self-diagnosed with orthorexia nervosa, potentially suggesting that the sample might not entirely reflect a true representation of orthorexia nervosa. Additionally, majority of the sample was female therefore the findings could potentially not be replicated in a male sample. This is the only study that has highlighted a link between parental expectations and orthorexia, therefore further explorations should be conducted in a sample that consists of individuals who score highly on diagnostic measures of orthorexia nervosa.

Additionally, the present study has conducted two mediations where self-compassion and mindfulness acted as mediators in the relationship between perfectionism and orthorexia nervosa. Both self-compassion and mindfulness were significant mediators, which is a novel finding in relation to orthorexia research. Based on the past literature both of these concepts have been successful mediators where perfectionism was one of the variables. For example, Manove and Khoury (2023) found that mindfulness was a successful mediator between perfectionism and societal anxiety, according to the diagnostic criteria distress or impairment of social functioning is a characteristic of orthorexia nervosa (Dunn & Bratman, 2016) Furthermore, mindfulness was also a successful mediator between perfectionism and negative thoughts (Short & Mazmanian, 2013), highlighting that mindfulness could be a significant mediator as orthorexic individuals experience negative thoughts when deviating from their dietary rules (Dunn & Bratman, 2016). Additionally, self-compassion was a successful mediator in the relationship between restrictive eating and orthorexia nervosa (Kalika et al., 2022). The significant mediating roles of self-compassion and mindfulness underscore their potential importance in understanding the mechanisms underlying orthorexia, and the alignment with prior research on perfectionism.

### Limitations

The present study has a number of limitations. There is a debate in terms of which orthorexia measure seems more viable. The current study has used the Orthorexia Nervosa Inventory (Oberle et al., 2020) which is the newest measure of orthorexia, therefore, has been used a limited amount in the research (Kalika et al., 2022; Kaya et al., 2021; Oberle et al., 2020). As previously discussed, there could be differences between mindfulness and self-compassion if other measures of orthorexia are used. For example, Kalika et al. (2023) demonstrated that there was no association between mindful eating and orthorexia when using the Dusseldorf Orthorexia Scale; however, Kalika et al. (2022) used the ONI which demonstrated a positive correlation with mindful eating. Therefore, future research into self-compassion and mindfulness should utilise other measures of orthorexia to establish if the findings are replicated. A prospective avenue for further exploration lies in examining the ramifications of healthy orthorexia and the dual capacity of mindfulness-based constructs to both foster and undermine manifestations of both constructive and detrimental forms of orthorexia.

A caution should be taken when interpreting the results due to the small number of participants in this study as well as ratio between the genders. Future studies should utilise higher sample size as well as making sure that the gender ratio is equal as these are important for the generalizability of the study.

### Future Directions

As demonstrated by the present study, self-compassion and mindfulness have a mediating capacity with orthorexia. Future research should look into experimental approaches utilising mindfulness and self-compassion-based interventions to determine their effectiveness in reducing orthorexic tendencies. Orthorexia nervosa has gained a lot of popularity with research looking at cross-sectional data, therefore utilising an experimental approach will add further insight into many much-needed interventions for orthorexia.

Furthermore, there is a need for qualitative research when it comes to mindfulness and self-compassion in the orthorexic population. Currently, there is limited literature that explored orthorexia qualitatively (e.g., Cheshire et al., 2020; Valente et al., 2020; White et al., 2021) and to date, no one looked at mindfulness, self-compassion and ON specifically. Gaining a deeper understanding of those concepts would allow further development of potential interventions for the orthorexic population.

### Conclusion

In conclusion, ON is positively correlated with perfectionism and negatively correlated to mindfulness and self-compassion. The present study has also conducted a mediation analysis which revealed that mindfulness and self-compassion can successfully mediate the relationship between perfectionism and orthorexia nervosa. The study offers a novel approach to understanding perfectionism with orthorexia, highlighting that self-compassion and mindfulness can be used as key components in much-needed interventions for orthorexia nervosa. Further research needs to explore these concepts further, especially experimentally and qualitatively as this would aid in further understanding of orthorexia nervosa.

## Supplemental Material

Supplemental Material - Exploration of the Mediating Role of Self-Compassion and Mindfulness on Orthorexia Nervosa and Perfectionism Supplemental Material for Exploration of the Mediating Role of Self-Compassion and Mindfulness on Orthorexia Nervosa and Perfectionism by Eliza Kalika, Misba Hussain, Helen Egan, Michail Mantzios in Psychological Reports

## Data Availability

The datasets generated during and/or analysed during the current study are available from the corresponding author on reasonable request. *

## References

[bibr1-00332941241256886] Awad E. Salameh P. Sacre H. Malaeb D. Hallit S. Obeïd S. (2021). Association between impulsivity and orthorexia nervosa/healthy orthorexia: Any mediating effect of depression, anxiety, and stress? BMC Psychiatry, 21(1). 10.1186/s12888-021-03594-4 PMC8640965 34861836

[bibr2-00332941241256886] Baer R. A. Smith G. T. Lykins E. L. B. Button D. F. Krietemeyer J. Sauer S. E. Walsh E. Duggan D. S. Williams J. M. G. (2008). Construct validity of the five facet mindfulness questionnaire in meditating and nonmeditating samples. Assessment, 15(3), 329–342. 10.1177/1073191107313003 18310597

[bibr3-00332941241256886] Barakat M. Salim N. A. Malaeb D. Dabbous M. Sakr F. Hallit S. Fekih-Romdhame F. Obeïd S. (2024). Mediating effect of psychological distress and mindful eating behaviors between orthorexia nervosa and academic self-efficacy among Lebanese university female students. BMC Public Health, 24(1), 136. 10.1186/s12889-024-17812-7 38308268 PMC10836016

[bibr4-00332941241256886] Bardone-Cone A. M. Sturm K. Lawson M. A. Robinson D. P. Smith R. (2009). Perfectionism across stages of recovery from eating disorders. International Journal of Eating Disorders, 43(2), 139–148. 10.1002/eat.20674 PMC2820585 19308994

[bibr5-00332941241256886] Bardone-Cone A. M. Wonderlich S. A. Frost R. O. Bulik C. M. Mitchell J. E. Uppala S. Simonich H. (2007). Perfectionism and eating disorders: Current status and future directions. Clinical Psychology Review, 27(3), 384–405. 10.1016/j.cpr.2006.12.005 17267086

[bibr7-00332941241256886] Barnes M. A. Caltabiano M. L. (2016). The interrelationship between orthorexia nervosa, perfectionism, body image and attachment style. Eating and Weight Disorders-studies on Anorexia Bulimia and Obesity, 22(1), 177–184. 10.1007/s40519-016-0280-x 27068175

[bibr8-00332941241256886] Bartel S. Sherry S. Farthing G. Stewart S. H. (2020). Classification of orthorexia nervosa: Further evidence for placement within the eating disorders spectrum. Eating Behaviors, 38, 101406. 10.1016/j.eatbeh.2020.101406 32540715

[bibr9-00332941241256886] Beshara M. Hutchinson A. D. Wilson C. (2013). Does mindfulness matter? Everyday mindfulness, mindful eating and self-reported serving size of energy dense foods among a sample of South Australian adults. Appetite, 67, 25–29. 10.1016/j.appet.2013.03.012 23548262

[bibr10-00332941241256886] Bohlmeijer E. T. Klooster P. M. T. Fledderus M. Veehof M. Baer R. A. (2011). Psychometric properties of the five facet mindfulness questionnaire in depressed adults and development of a short form. Assessment, 18(3), 308–320. 10.1177/1073191111408231 21586480

[bibr11-00332941241256886] Boisseau C. L. Thompson-Brenner H. Pratt E. M. Farchione T. J. Barlow D. H. (2013). The relationship between decision-making and perfectionism in obsessive-compulsive disorder and eating disorders. Journal of Behavior Therapy and Experimental Psychiatry, 44(3), 316–321. 10.1016/j.jbtep.2013.01.006 23454627

[bibr12-00332941241256886] Bratman S. (2017). Orthorexia vs. theories of healthy eating. Eating and Weight Disorders - Studies on Anorexia, Bulimia and Obesity, 22(3), 381–385. 10.1007/s40519-017-0417-6 28741285

[bibr13-00332941241256886] Braun T. D. Park C. L. Gorin A. A. (2016). Self-compassion, body image, and disordered eating: A review of the literature. Body Image, 17, 117–131. 10.1016/j.bodyim.2016.03.003 27038782

[bibr14-00332941241256886] Brown A. R. Parman K. M. Rudat D. A. Craighead L. W. (2012). Disordered eating, perfectionism, and food rules. Eating Behaviors, 13(4), 347–353. 10.1016/j.eatbeh.2012.05.011 23121786

[bibr15-00332941241256886] Brytek-Matera A. Fonte M. L. Poggiogalle E. Donini L. M. Cena H. (2017). Orthorexia nervosa: Relationship with obsessive-compulsive symptoms, disordered eating patterns and body uneasiness among Italian university students. Eating and Weight Disorders-Studies on Anorexia, Bulimia and Obesity, 22(4), 609–617. 10.1007/s40519-017-0427-4 28840493

[bibr16-00332941241256886] Bulik C. M. Tozzi F. Anderson C. B. Mazzeo S. E. Aggen S. H. Sullivan P. F. (2003). The relation between eating disorders and components of perfectionism. American Journal of Psychiatry, 160(2), 366–368. 10.1176/appi.ajp.160.2.366 12562586

[bibr17-00332941241256886] Cena H. Barthels F. Cuzzolaro M. Bratman S. Brytek-Matera A. Dunn T. Varga M. Missbach B. Donini L. M. (2018). Definition and diagnostic criteria for orthorexia nervosa: A narrative review of the literature. Eating and Weight Disorders - Studies on Anorexia, Bulimia and Obesity, 24(2), 209–246. 10.1007/s40519-018-0606-y 30414078

[bibr18-00332941241256886] Cheshire A. Berry M. Fixsen A. (2020). What are the key features of orthorexia nervosa and influences on its development? A qualitative investigation. Appetite, 155, 104798. 10.1016/j.appet.2020.104798 32717291

[bibr19-00332941241256886] Cohen J. (1992). Statistical power analysis. Current Directions in Psychological Science, 1(3), 98–101. 10.1111/1467-8721.ep10768783

[bibr20-00332941241256886] Davies H. Liao P. Campbell I. C. Tchanturia K. (2009). Multidimensional self reports as a measure of characteristics in people with eating disorders. Eating and Weight Disorders-studies on Anorexia Bulimia and Obesity, 14(2–3), e84–e91. 10.1007/bf03327804 19934641

[bibr21-00332941241256886] Donini L. M. Marsili D. Graziani M. P. Imbriale M. Cannella C. (2004). Orthorexia nervosa: A preliminary study with a proposal for diagnosis and an attempt to measure the dimension of the phenomenon. Eating and Weight Disorders - Studies on Anorexia, Bulimia and Obesity, 9(2), 151–157. 10.1007/bf03325060 15330084

[bibr22-00332941241256886] Dunn T. M. Bratman S. (2016). On orthorexia nervosa: A review of the literature and proposed diagnostic criteria. Eating Behaviors, 21, 11–17. 10.1016/j.eatbeh.2015.12.006 26724459

[bibr23-00332941241256886] Dutt S. Keyte R. Egan H. Hussain M. Mantzios M. (2019). Healthy and unhealthy eating amongst stressed students: Considering the influence of mindfulness on eating choices and consumption. Health Psychology Report, 7(2), 113–120. 10.5114/hpr.2019.77913

[bibr24-00332941241256886] Egan S. J. Wade T. D. Shafran R. (2011). Perfectionism as a transdiagnostic process: A clinical review. Clinical Psychology Review, 31(2), 203–212. 10.1016/j.cpr.2010.04.009 20488598

[bibr25-00332941241256886] Ferreira C. Pinto-Gouveia J. Duarte C. (2014). Self-criticism, perfectionism and eating disorders: The effect of depression and body dissatisfaction. International Journal of Psychology and Psychological Therapy, 14(3), 409–420.

[bibr26-00332941241256886] Flett G. L. Nepon T. Hewitt P. L. Rose A. (2020). Why perfectionism is antithetical to mindfulness: A conceptual and empirical analysis and consideration of treatment implications. International Journal of Mental Health and Addiction, 19(5), 1625–1645. 10.1007/s11469-020-00252-w

[bibr27-00332941241256886] Franco-Paredes K. Díaz J. M. M. Arévalo R. V. Aguilar X. L. Alvarez-Rayón G. (2004). Perfectionism and eating disorders: A review of the literature. European Eating Disorders Review, 13(1), 61–70. 10.1002/erv.605

[bibr28-00332941241256886] Fritz M. S. MacKinnon D. P. (2007). Required sample size to detect the mediated effect. Psychological Science, 18(3), 233–239. 10.1111/j.1467-9280.2007.01882.x 17444920 PMC2843527

[bibr29-00332941241256886] Frost R. O. Marten P. A. Lahart C. Rosenblate R. (1990). The dimensions of perfectionism. Cognitive Therapy and Research, 14(5), 449–468. 10.1007/bf01172967

[bibr30-00332941241256886] Goldstein M. Peters L. Thornton C. Touyz S. (2014). The treatment of perfectionism within the eating disorders: A pilot study. European Eating Disorders Review, 22(3), 217–221. 10.1002/erv.2281 24474602

[bibr31-00332941241256886] Hair J. Black W. C. Babin B. J. Anderson R. E. (2010). Multivariate data analysis (7th ed.). Pearson Educational International.

[bibr32-00332941241256886] Hanganu-Bresch C. (2019). Orthorexia: Eating right in the context of healthism. Medical Humanities, 46(3), 311–322. 10.1136/medhum-2019-011681 31358564

[bibr33-00332941241256886] Hewitt P. L. Flett G. L. Turnbull-Donovan W. Mikail S. F. (1991). The Multidimensional Perfectionism Scale: Reliability, validity, and psychometric properties in psychiatric samples. Psychological Assessment, 3(3), 464–468. 10.1037/1040-3590.3.3.464

[bibr34-00332941241256886] James K. Rimes K. A. (2017). Mindfulness-based cognitive therapy versus pure cognitive behavioural self-help for perfectionism: A pilot randomised study. Mindfulness, 9(3), 801–814. 10.1007/s12671-017-0817-8 29875882 PMC5968046

[bibr36-00332941241256886] Kabat-Zinn J. (2003). Mindfulness-based interventions in context: Past, present, and future. Clinical Psychology: Science and Practice, 10(2), 144–156. 10.1093/clipsy.bpg016

[bibr37-00332941241256886] Kalika E. Egan H. Mantzios M. (2022). Exploring the role of mindful eating and self-compassion on eating behaviours and orthorexia in people following a vegan diet. Eating and Weight Disorders - Studies on Anorexia, Bulimia and Obesity, 27, 2641–2651. 10.1007/s40519-022-01407-5 PMC9556376 35553382

[bibr38-00332941241256886] Kalika E. Hussain M. Egan H. Mantzios M. (2023). Exploring the moderating role of mindfulness, mindful eating, and self-compassion on the relationship between eating-disordered quality of life and orthorexia nervosa. Eating and Weight Disorders-studies on Anorexia Bulimia and Obesity, 28(1). 10.1007/s40519-023-01542-7 PMC9941235 36808014

[bibr39-00332941241256886] Kaya S. Uzdil Z. Çakıroğlu F. P. (2021). Validation of the Turkish version of the Orthorexia Nervosa Inventory (ONI) in an adult population: Its association with psychometric properties. Eating and Weight Disorders - Studies on Anorexia, Bulimia and Obesity, 27(2), 729–735. 10.1007/s40519-021-01199-0 34028783

[bibr40-00332941241256886] Koven N. Abry A. (2015). The clinical basis of orthorexia nervosa: Emerging perspectives. Neuropsychiatric Disease and Treatment, 11, 385. 10.2147/ndt.s61665 25733839 PMC4340368

[bibr41-00332941241256886] Kuyken W. Watkins E. Holden E. White K. Taylor R. S. Byford S. Evans A. Radford S. Teasdale J. D. Dalgleish T. (2010). How does mindfulness-based cognitive therapy work? Behaviour Research and Therapy, 48(11), 1105–1112. 10.1016/j.brat.2010.08.003 20810101

[bibr42-00332941241256886] Lewthwaite M. LaMarre A. (2022). “That’s just healthy eating in my opinion” - Balancing understandings of health and ‘orthorexic’ dietary and exercise practices. Appetite, 171, 105938. 10.1016/j.appet.2022.105938 35066005

[bibr43-00332941241256886] Manova V. Khoury B. (2023). Interpersonal perfectionism and social anxiety: The mediational role of mindfulness. Canadian Journal of Behavioural Science. Online first publication. 10.1037/cbs0000370

[bibr44-00332941241256886] Mantzios M. Egan H. Asif T. (2019). A randomised experiment evaluating the mindful raisin practice as a method of reducing chocolate consumption during and after a mindless activity. Journal of Cognitive Enhancement, 4(3), 250–257. 10.1007/s41465-019-00159-y

[bibr45-00332941241256886] Mantzios M. Egan H. Bahia H. Hussain M. Keyte R. (2018). How does grazing relate to body mass index, self-compassion, mindfulness and mindful eating in a student population? Health Psychology Open, 5(1), 205510291876270. 10.1177/2055102918762701 PMC5846935 29552351

[bibr46-00332941241256886] Mantzios M. Wilson J. C. (2013). Making concrete construals mindful: A novel approach for developing mindfulness and self-compassion to assist weight loss. Psychology & Health, 29(4), 422–441. 10.1080/08870446.2013.863883 24215123

[bibr47-00332941241256886] Mantzios M. Wilson J. C. (2014). Exploring mindfulness and mindfulness with self-compassion-centered interventions to assist weight loss: Theoretical considerations and preliminary results of a randomized pilot study. Mindfulness, 6(4), 824–835. 10.1007/s12671-014-0325-z

[bibr48-00332941241256886] Mantzios M. Wilson J. C. (2015). Mindfulness, eating behaviours, and obesity: A review and reflection on current findings. Current Obesity Reports, 4(1), 141–146. 10.1007/s13679-014-0131-x 26627097

[bibr49-00332941241256886] Mathieu J. (2005). What is orthorexia? Journal of the American Dietetic Association, 105(10), 1510–1512. 10.1016/j.jada.2005.08.021 16183346

[bibr50-00332941241256886] McComb S. E. Mills J. S. (2019). Orthorexia nervosa: A review of psychosocial risk factors. Appetite, 140, 50–75. 10.1016/j.appet.2019.05.005 31075324

[bibr51-00332941241256886] Merhy G. Moubarak V. Hallit R. Obeid S. Hallit S. (2023). The indirect role of orthorexia nervosa and eating attitudes in the association between perfectionism and muscle dysmorphic disorder in Lebanese male University students – results of a pilot study. BMC Psychiatry, 23(1), 104. 10.1186/s12888-023-04549-7 36670380 PMC9854036

[bibr52-00332941241256886] Messer M. Liu C. Linardon J. (2023). Orthorexia nervosa symptoms prospectively predict symptoms of eating disorders and depression. Eating Behaviors, 49, 101734. 10.1016/j.eatbeh.2023.101734 37146411

[bibr53-00332941241256886] Miley M. Egan H. Wallis D. J. Mantzios M. (2022). Orthorexia nervosa, mindful eating, and perfectionism: An exploratory investigation. Eating and Weight Disorders-studies on Anorexia Bulimia and Obesity, 27(7), 2869–2878. 10.1007/s40519-022-01440-4 PMC9556414 35829900

[bibr54-00332941241256886] Neff K. (2003a). Self-Compassion: An alternative conceptualization of a healthy attitude toward oneself. Self and Identity, 2(2), 85–101. 10.1080/15298860309032

[bibr55-00332941241256886] Neff K. D. (2003b). The development and validation of a scale to measure Self-Compassion. Self and Identity, 2(3), 223–250. 10.1080/15298860309027

[bibr56-00332941241256886] Neff K. D. (2015). The self-compassion scale is a valid and theoretically coherent measure of self-compassion. Mindfulness, 7(1), 264–274. 10.1007/s12671-015-0479-3

[bibr57-00332941241256886] Neff K. D. Kirkpatrick K. L. Rude S. S. (2007). Self-compassion and adaptive psychological functioning. Journal of Research in Personality, 41(1), 139–154. 10.1016/j.jrp.2006.03.004

[bibr83-00332941241256886] Neff K. D. Knox M. (2017). Self-Compassion. In Springer eBooks, 1–8. 10.1007/978-3-319-28099-8_1159-1

[bibr58-00332941241256886] Neff K. D. Pommier E. (2013). The relationship between self-compassion and other-focused concern among college undergraduates, community adults, and practicing meditators. Self and Identity, 12(2), 160–176. 10.1080/15298868.2011.649546

[bibr59-00332941241256886] Novara C. Mattioli S. Piasentin S. Pardini S. Maggio E. (2022). The role of dieting, psychopathological characteristics and maladaptive personality traits in Orthorexia Nervosa. BMC Psychiatry, 22(1), 290. 10.1186/s12888-022-03896-1 35459152 PMC9034604

[bibr60-00332941241256886] Novara C. Pardini S. Maggio E. Mattioli S. Piasentin S. (2021). Orthorexia Nervosa: Over concern or obsession about healthy food? Eating and Weight Disorders-studies on Anorexia Bulimia and Obesity, 26(8), 2577–2588. 10.1007/s40519-021-01110-x PMC8602217 33566324

[bibr61-00332941241256886] Oberle C. D. De Nadai A. S. Madrid A. L. (2020). Orthorexia nervosa inventory (ONI): Development and validation of a new measure of orthorexic symptomatology. Eating and Weight Disorders - Studies on Anorexia, Bulimia and Obesity, 26, 609–622. 10.1007/s40519-020-00896-6 32279201

[bibr62-00332941241256886] Pratt V. B. Hill A. P. Madigan D. J. (2023). A longitudinal study of perfectionism and orthorexia in exercisers. Appetite, 183, 106455. 10.1016/j.appet.2023.106455 36623773

[bibr63-00332941241256886] Preacher K. J. Hayes A. F. (2008). Asymptotic and resampling strategies for assessing and comparing indirect effects in multiple mediator models. Behavior Research Methods, 40(3), 879–891. 18697684 10.3758/brm.40.3.879

[bibr64-00332941241256886] Rand-Giovannetti D. Rozzell K. N. Latner J. D. (2022). The role of positive self-compassion, distress tolerance, and social problem-solving in the relationship between perfectionism and disordered eating among racially and ethnically diverse college students. Eating Behaviors, 44, 101598. 10.1016/j.eatbeh.2022.101598 35149442

[bibr65-00332941241256886] Ratner B. (2009). The correlation coefficient: Its values range between +1/−1, or do they? Journal of Targeting, Measurement and Analysis for Marketing, 17(2), 139–142. 10.1057/jt.2009.5

[bibr66-00332941241256886] Robinson K. A. Wade T. D. (2021). Perfectionism interventions targeting disordered eating: A systematic review and meta-analysis. International Journal of Eating Disorders, 54(4), 473–487. 10.1002/eat.23483 33594679

[bibr67-00332941241256886] Roncero M. Barrada J. R. García-Soriano G. Guillén V. (2021). Personality profile in orthorexia nervosa and healthy orthorexia. Frontiers in Psychology, 12, 710604. 10.3389/fpsyg.2021.710604 34594274 PMC8477971

[bibr69-00332941241256886] Short M. Mazmanian D. (2013). Perfectionism and negative repetitive thoughts: Examining a multiple mediator model in relation to mindfulness. Personality and Individual Differences, 55(6), 716–721. 10.1016/j.paid.2013.05.026

[bibr84-00332941241256886] Siegel R. D. Germer C. K. Olendzki A. (2009). Mindfulness: What Is It? Where Did It Come From? In Didonna, F. (eds) Clinical Handbook of Mindfulness. New York: Springer.

[bibr70-00332941241256886] Stöber J. (1998). The Frost Multidimensional Perfectionism Scale revisited: More perfect with four (instead of six) dimensions. Personality and Individual Differences, 24(4), 481–491. 10.1016/s0191-8869(97)00207-9

[bibr71-00332941241256886] Strahler J. (2020). Trait mindfulness differentiates the interest in healthy diet from orthorexia nervosa. Eating and Weight Disorders - Studies on Anorexia, Bulimia and Obesity, 26(3). 10.1007/s40519-020-00927-2 PMC8004484 32445115

[bibr72-00332941241256886] Tabachnick B. G. Fidell L. S. (2007). Using multivariate statistics (7th edition). Pearson.

[bibr73-00332941241256886] Tobin R. Dunkley D. M. (2021). Self-critical perfectionism and lower mindfulness and self-compassion predict anxious and depressive symptoms over two years. Behaviour Research and Therapy, 136, 103780. 10.1016/j.brat.2020.103780 33259957

[bibr74-00332941241256886] Turner P. G. Lefevre C. E. (2017). Instagram use is linked to increased symptoms of orthorexia nervosa. Eating and Weight Disorders-studies on Anorexia Bulimia and Obesity, 22(2), 277–284. 10.1007/s40519-017-0364-2 PMC5440477 28251592

[bibr75-00332941241256886] Valente M. Brenner R. Cesuroglu T. Bunders-Aelen J. Syurina E. V. (2020). “And it snowballed from there”: The development of orthorexia nervosa from the perspective of people who self-diagnose. Appetite, 155, 104840. 10.1016/j.appet.2020.104840 32822807

[bibr76-00332941241256886] White M. Berry R. Sharma A. Rodgers R. F. (2021). A qualitative investigation of Orthorexia Nervosa among U.S. college students: Characteristics and sociocultural influences. Appetite, 162, 105168. 10.1016/j.appet.2021.105168 33617934

[bibr77-00332941241256886] Yung J. J. Tabri N. (2022). The association of perfectionism, health‐focused self‐concept, and erroneous beliefs with orthorexia nervosa symptoms: A moderated mediation model. International Journal of Eating Disorders, 55(7), 892–901. 10.1002/eat.23719 35514117

[bibr78-00332941241256886] Zagaria A. Barbaranelli C. Mocini E. Lombardo C. (2023). Cross-cultural adaptation and psychometric properties of the Italian version of the Orthorexia Nervosa Inventory (ONI). Journal of Eating Disorders, 11(1), 11. 10.1186/s40337-023-00858-0 37620907 PMC10463941

[bibr79-00332941241256886] Zickgraf H. F. Barrada J. R. (2021). Orthorexia nervosa vs. healthy orthorexia: Relationships with disordered eating, eating behavior, and healthy lifestyle choices. Eating and Weight Disorders-studies on Anorexia Bulimia and Obesity, 27(4), 1313–1325. 10.1007/s40519-021-01263-9 PMC8286169 34275120

