# Supplementary material for: Exploration of the Mediating Role of Self-Compassion and Mindfulness on Orthorexia Nervosa and Perfectionism
Source: Psychol Rep. 2024 Jun 4;129(2):1202–21. doi: 10.1177/00332941241256886 (PMC12953670; doi:10.1177/00332941241256886)
Supplement: Supplemental Material - Exploration of the Mediating Role of Self-Compassion and Mindfulness on Orthorexia Nervosa and Perfectionism [file sj-pdf-1-prx-10.1177_00332941241256886.pdf]

Supplementary materials

Table 2.

Bivariate correlations between subscales of ONI, SCS, FFMQ, SDHS and FMPS and descriptive statistics.

	1	*2*	*3*	*4*	*5*	*M*	*SD*
(1) Behaviour (ONI)						*18.46*	*7.53*
(2) Emotion (ONI)	*.818***					*10.70*	*4.42*
(3) Impairments (ONI)	*.822***	*.788***				*17.50*	*8.36*
(4) FFMQ	*-358***	*-.397***	*-.441***			*45.57*	*7.35*
(5) SCS	*-.401***	*-.507***	*-.442***	*.663***		*2.74*	*0.84*
(6) SDHS	*.403***	*.488***	*.494***	*-.382***	*-.648***	*94.40*	*23.80*

Note: ONI: Orthorexia Nervosa Inventory. FFMQ: Five-Facet Mindfulness Questionnaire SCS: Self-Compassion Scale. FMPS: Frost Multidimensional Perfectionism

*Correlation is significant at the .05 level

**Correlation is significant at the .01 level

Table 3.

Bivariate correlations between ONI and subscales of FFMQ, SCS and FMPS.

	1	*2*	*3*	*4*	*5*	*6*	*7*	*8*	*9*	*10*	*11*	*12*	*13*	*15*	*15*	*M*	*SD*
(1) ONI																*46.66*	*19.05*
(2)Perf Mistakes	*.543***															*41.18*	*11.84*
(3) Perf Expec	*.419***	*.688***														*25.64*	*9.67*
(4) Perf High Standards	*.244***	*.662***	*.507***													*25.15*	*5.62*
(5) Perf Organisation	*.148*	*.206**	*.207**	*.387***												*22.01*	*4.96*
(6) SCS Kindness	*-.234***	*-.400***	*-.271***	*.-299***	*.004*											*2.22*	*0.98*
(7)SCS Judgement	*-.369***	*-.663***	*-.461***	*-.516***	*-.200**	*.555***										*2.97*	*1.16*
(8)SCS Humanity	*-.296***	*-.411***	*-.280***	*-.201**	*.098*	*.600***	*.383***									*2.38*	*1.04*
(9)SCS Isolation	*-.487***	*-.716***	*-.493***	*-.437***	*-.185**	*.427***	*.755***	*.361***								*3.18*	*1.19*
(10)SCS Mindfulness	*-.200**	*-.356***	*-.295***	*-.137*	*.023*	*.727***	*.391***	*.657***	*.360***							*3.61*	*1.01*
(11)SCS Identification	*-.452***	*-.674***	*-.427***	*-.393***	*-.091*	*.345***	*.749***	*.290***	*.735***	*.318***						*3.07*	*1.07*

(12)FFMQ Obser	*.191**	*.098*	*.177**	*.111*	*.115*	*.214**	*-.016*	*.158*	*-.106*	*.177**	*-.071*					*9.35*	*2.72*
(13)FFMQ Descrip	*-.258***	*-.091*	*-.086*	*.115*	*.137*	*.108*	*.124*	*.104*	*.117*	*.052*	*.136*	*.062*				*9.40*	*2.81*
(14)FFMQ Awareness	*-.285***	*-.355***	*-.300***	*.000*	*.181**	*.071*	*.268***	*.164*	*.358***	*.123*	*.300***	*.042*	*.126*			*8.99*	*2.66*
(15)FFMQ Judgement	*-.544***	*-.651***	*-.482***	*-.321***	*-.137*	*.370***	*.594***	*.335***	*.675***	*.388***	*.553***	*-.189**	*.109*	*.373***		*9.31*	*3.28*
(16)FFMQ React	*-.147*	*-.129*	*-.053*	*-.094*	*.055*	*.400***	*.407***	*.394***	*.290***	*.441***	*.341***	*.096*	*.172**	*-.084*	*.075*	*8.53*	*2.59*

Note: ONI: Orthorexia Nervosa Inventory. BMI: Body Mass Index. FFMQ: Five-Facet Mindfulness Questionnaire SCSd: Self-Compassion Scale. FMPS: Frost Multidimensional Perfectionism Scale.
*Correlation is significant at the .05 level
**Correlation is significant at the .01 level
